# Children’s memory “in the wild”: examining the temporal organization of free recall from a week-long camp at a local zoo

**DOI:** 10.1186/s41235-022-00452-z

**Published:** 2023-01-25

**Authors:** Thanujeni Pathman, Lina Deker, Puneet Kaur Parmar, Mark Christopher Adkins, Sean M. Polyn

**Affiliations:** 1 Department of Psychology, York University, 4700 Keele Street, Toronto, ON M3J 1P3 Canada; 2 Department of Psychology, Vanderbilt University, Nashville, TN USA

**Keywords:** Episodic memory, Free recall, Temporal clustering, Children, Naturalistic, Real-world events, Memory development

## Abstract

Free-recall paradigms have greatly influenced our understanding of memory. The majority of this research involves laboratory-based events (e.g., word lists) that are studied and tested within minutes. This literature shows that adults recall events in a temporally organized way, with successive responses often coming from neighboring list positions (i.e., temporal clustering) and with enhanced memorability of items from the end of a list (i.e., recency). Temporal clustering effects are so robust that temporal organization is described as a fundamental memory property. Yet relatively little is known about the development of this temporal structure across childhood, and even less about children’s memory search for real-world events occurring over an extended period. In the present work, children (*N* = 144; 3 age groups: 4–5-year-olds, 6–7-year-olds, 8–10-year-olds) took part in a 5-day summer camp at a local zoo. The camp involved various dynamic events, including daily animal exhibit visits. On day 5, children were asked to recall all the animals they visited. We found that overall recall performance, in terms of number of animals recalled, improved steadily across childhood. Temporal organization and recency effects showed different developmental patterns. Temporal clustering was evident in the response sequences for all age groups and became progressively stronger across childhood. In contrast, the recency advantage, when characterized as a proportion of total responses, was stable across age groups. Thus, recall dynamics in early childhood parallel that seen in adulthood, with continued development of temporal organization across middle to late childhood.

**Supplementary Information:**

The online version contains supplementary material available at 10.1186/s41235-022-00452-z.

The human memory system undergoes dramatic development in early to late childhood. With increasing age, children correctly recall more items, like words or pictures (e.g., Glidden, 1977; Kobasigawa & Middleton, 1972; Waber et al., 2007), remember events over longer delays (Ornstein et al., 1992), provide more detailed and coherent accounts of past experiences (Reese et al., 2011) and show differential use of explicit memory strategies (see Bjorklund et al., 2009; Schneider & Ornstein, 2015). This work has been foundational to our understanding of memory development (see Bauer & Fivush, 2014). Still, there is much to learn about how the developing memory system matures into the adult system, particularly with regards to memory search.

Decades of research using the laboratory-based free-recall paradigm has revealed that there are underlying principles that guide adult memory search (Healey & Kahana, 2014; see reviews Kahana, 2020; Polyn & Kahana, 2008; Raaijmakers & Shiffrin, 1981). In a typical free-recall paradigm, participants study a list of items (like words) and are then asked to recall as many items as possible. Examination of the order in which items are recalled (i.e., the transitions between subsequent items in participant responses) reveals reliable organizational effects that reflect how adults search through and retrieve events from our episodic memory system: temporal clustering (items that are experienced closer in time tend to be recalled consecutively; Kahana, 1996), spatial clustering (items or landmarks that are geographically closer to each other tend to be recalled consecutively; Miller et al., 2013a, 2013b) and semantic clustering (items that are meaningfully related tend to be recalled consecutively; Manning & Kahana, 2012). Temporal contiguity effects, the majority of research based on temporal clustering, are so robust that researchers call it a fundamental property of our memory system (Healey & Kahana, 2014; Healey et al., 2019; Kelly & Beran, 2021). These effects are theoretically meaningful, as individual differences in the strength of temporal clustering are positively correlated with recall performance (Sederberg, et al., 2010; see Healey et al., 2014, 2019; Spillers & Unsworth, 2011). Here, we examine the development of temporal clustering and whether this principle extends to naturally occurring, real-world situations in children.

Few studies with children have examined temporal clustering effects and how this clustering may differ across childhood. The youngest group tested, 4–5-year-olds, showed “a small tendency” (p. 837) to transition to a neighboring item in a picture recall task (Kelly & Beran, 2021). In older children, Lehmann and Hasselhorn (2010; see also Lehmann & Hasselhorn, 2012) assessed immediate recall of word lists in 8-year-olds, testing children on five occasions over two years, up until age 10. When recalling an item, children tended to follow this recall with an item from a nearby list position, with adjacent-item temporal clustering becoming more pronounced as children grew older. Jarrold and colleagues (2015) found that 5–6- and 7–8-year-olds showed evidence of temporal clustering during immediate recall; however, they did not find differences in the degree of temporal clustering between age groups. This literature using laboratory-based immediate free recall provides us with important indications about when temporal clustering effects emerge and how effects possibly change as children grow older. However, additional studies are needed to resolve mixed findings about developmental change. Further, no studies to our knowledge have assessed temporal clustering using naturalistic events in children.

A handful of studies have examined temporal organization of free recall using autobiographical or naturalistic events (see review, Healey et al., 2019). Adults showed temporal clustering when freely recalling news stories (they were more likely to successively recall stories that occurred within a few days of each other than would be expected by chance; Uitvlugt & Healey, 2019) and when recalling personal autobiographical events (Moreton & Ward, 2010). Diamond and Levine (2020) asked younger and older adults to take part in an audio-guided walking tour lasting 20–30 min. After 2 days, participants were asked to tell researchers everything they remembered about the tour and researchers examined recall narratives to determine the order of recall for tour elements. Both younger and older adults showed temporal clustering effects such that recall transitions were more likely to be between nearby tour elements. Further, although both groups showed above chance levels of temporal clustering, younger adults showed more clustering than older adults.

These studies extend laboratory-based temporal clustering effects to naturalistic events and parameters, such that there are other naturally occurring events before and after each study item and also after the last study item. This is similar to continual-distractor free recall, a version of the free-recall paradigm where the participant performs a distraction task before and after each study item (e.g., Bjork & Whitten, 1974; Koppenaal & Glanzer, 1990; see Kahana, 2020). This work also suggests that, at least later in the lifespan, examining the temporal organization of recall in the real world can be used to document age-related differences in memory search.

In naturalistic studies (e.g., Diamond & Levine, 2020), participants move about in space as they move forward in time. In the laboratory, one can potentially disentangle the influences of spatial position and temporal location (e.g., Miller et al., 2013a, 2013b; Miller et al., 2013a, 2013b). The clustering observed in naturalistic studies might be more accurately described as spatiotemporal clustering, but for the sake of continuity with the broader literature, we refer to it as temporal clustering. (We revisit this in the Discussion.)

The primary goal of the present study was to extend past laboratory-based and naturalistic free-recall studies by testing whether temporal clustering effects are observed in childhood using naturalistic and dynamic events and parameters, namely a multi-day sequence of naturally occurring events of which a subset of events are later freely recalled. We applied analytic tools from traditional list-learning studies to test when the executive processes that give rise to temporal clustering become functional, and whether there are age-related differences in the strength of temporal clustering. Thus, our study will allow us to test the universality of temporal clustering effects in children (i.e., will the effects previously found with word or picture lists be found outside the laboratory?) and help to clarify the mixed findings about age group differences in the existing laboratory-based research on childhood temporal clustering. A secondary goal was to examine primacy and recency effects (e.g., Glidden, 1977) in memory for naturalistic events. Understanding memory search across childhood is necessary to test whether principles demonstrated in younger and older adults generalize to younger populations, and to build a comprehensive theoretical model of memory across the lifespan. Further, the present study is an attempt to answer separate calls for everyday memory studies to complement a century of laboratory-based studies (Cohen & Conway, 2007) and developmental research in the “messiness of the real world” (Golinkoff et al., 2017, p. 1407).

## Method

### Participants

Participants were children who took part in a 5-day (Monday to Friday; 9 am to 3:30 pm) summer camp at a local zoo in Canada. A total of 144 children participated: forty-five 4–5-year-olds (*M* = 5.20, SD = 0.53), forty-seven 6–7-year-olds (*M* = 6.97, SD = 0.58) and fifty-two 8–10-year-olds (*M* = 9.11, SD = 0.74). Based on demographic information provided by parents, eighty-one participants were female and sixty-three participants were male. The ethnicity of the participants in this study was 54.17% White or Caucasian, 20.14% Asian, 6.94% Asian and White or Caucasian, 3.47% West Indian and White or Caucasian, 2.78% Black and White or Caucasian, 2.78% Latin American, 2.08% Aboriginal or First Nations and White or Caucasian, 1.39% Black or African American or Canadian, 1.39% Aboriginal or First Nations, 1.39% Asian and Black or African American and White or Caucasian, 0.69% East Indian and White or Caucasian and 2.78% did not report an ethnicity. The family income reported by parents of participants was as follows: 0.69% less than $15,000, 1.39% between $15,000 and $40,000, 3.47% between $40,000 and $60,000, 12.50% between $60,000 and $90,000, 22.92% between $90,000 and $120,000, 52.08% more than $120,000 and 6.94% did not report an income.

Recruitment of participants occurred via an email sent to parents who had registered their child for the week-long camp during the months of July and August 2018. Parents provided online parental consent (prior to the testing session which occurred on Fridays). Children provided verbal assent before beginning the testing session. Procedures were approved by a university research ethics board. At the end of the study, children were given a “Junior Scientist Certificate.” In addition, parents were entered in a drawing for a free family membership to the local zoo. An additional 2 children began the testing session, but were not included in this sample because they asked to leave the testing session early (*n* = 1), or because there was not enough time to complete the task due to the next camp activity (*n* = 1). Two children in our sample participated a second time later in the summer; the second instance of participation was not included.

A maximum sample size for each age group was not predetermined. Our goal was to include as many participants as possible in this study. The sample size obtained was based on two constraints: the number of parents that completed the online consent form and the number of test sessions that could be completed within the finite time available to us for testing (see “Testing Session”). We note that the per group sample size is comparable or exceeds past research on children’s free recall and temporal organization.

## Procedure

### Learning phase

During the 5-day camp, children visited animal exhibits, engaged in fun activities and hands-on demonstrations, attended musical performances, and were taught facts about animals, conservation and other related topics from camp counsellors, zookeepers and other zoo staff. The zoo provided us with predetermined schedules that included information about which locations (i.e., exhibit areas) in the zoo the child would visit and in what order. For example, a child may go to the “Indomalaya” location from 9:30 pm to 11:00 pm, and the “Savanna” location from 12:30 pm to 2:30 pm. In addition, we knew which animals were in particular locations at the zoo. For example, we knew that the royal python, spotted-necked otter and pygmy hippopotamus were all located in the “Rainforest” location. If any changes were made to the daily schedule (e.g., due to weather or closing of an exhibit), camp counsellors noted the changes on a form provided by us. Thus, the predetermined schedules and camp counsellor checklists allowed us to determine when each participant visited particular locations of the zoo (i.e., when during the week they visited particular animals).

Across all weeks of the summer, camp schedules varied depending on the camp group and week. There were multiple camp groups per age, each of which had different schedules. Further, the schedules varied for camp groups across weeks. As a result, a location within the zoo that one 5-year-old participant visited on Wednesday, for example, another 5-year-old camper could have visited on Monday. In summary, particular animals visited on particular days, varied both within and across age groups.

The three age groups used in this study (4–5, 6–7 and 8–10-year-olds) were determined a priori. We note that the Zoo set the age of children based on birth year; child’s birth year (e.g., 2012) determined which of 3 camp groups (“Zoo Kids,” “Explorers,” “Adventurers”) the child was eligible to attend. We note that the ages of children in the 3 camp groups did not necessarily correspond to the 3 age groups used in this study. Further, within any particular camp group (e.g., Zoo Kids) there were different themes set by the Zoo for that particular week in the summer (e.g., “Zoo Clues,” “Growing up Wild,” “Eye Spy”). There were also multiple subgroups led by different counsellors for every camp group that week (e.g., “Zoo Kids 1,” “Zoo Kids 2,” etc.). Our study’s 4–5-year-old age group consisted of children who were in one of the “Zoo Kids” camp groups or one of the “Explorers” camp groups. Our study’s 6–7-year-old age group consisted of children who were in one of the “Explorers” camp groups or “Adventurers” camp groups. Our study’s 8–10-year-old age group consisted of children who were in one of the “Adventurers” camp groups.

To get a sense of the variability of camp peers and counsellors, we can look at our 8–10-year-olds further. There were 5 8–10-year-olds in “week 3” (these children split among 3 different subgroups with different counsellors leading each subgroup), 11 in “week 4” (split among 3 subgroups), 6 in “week 5” (split among 2 subgroups), 6 in “week 6” (split among 3 subgroups), 5 in “week 7” (split among 2 subgroups), 6 in “week 8” (split among 3 subgroups), 13 in “week 9” (split among 3 subgroups).

Zoo designated “Zoo Kids” camp groups (differing by week and theme; multiple subgroups led by different counsellors) included 4- and 5-year-old children, Zoo designated “Explorers” included 5-, 6- and 7-year-olds, and “Adventurers” included 7-, 8-, 9- and 10-year-olds. Thus, a 7-year-old in one of the “Explorers” groups would have a similar experience (in terms of sequence of activities and information provided by counsellors) as a 5–6-year-old child, while a 7-year-old in one of the “Adventurers” groups would have a similar experience to an 8–10-year-old child. In summary, camp peers and counsellors varied within age groups.

### Testing session

On the last day of zoo camp (i.e., a Friday), children participated in a short testing session (*M* = 14.31 min, SD = 4.98 min) where they were asked questions about their experience during the week at zoo camp. Children were tested individually during “downtime” after lunch, selected by the local zoo as a time which would not disrupt planned camp activities. Children were tested by one of seven female experimenters. The session began with a “warm up” question (“How much do you like learning about animals?”), and a question in which they were asked to describe one animal visit of their choosing (“What was your favorite animal you saw this week?”) if time allowed. The focus of this study was on a portion of the testing session that assessed children’s memory using free recall. Children were asked the following question by the experimenter, “You met so many different and cool animals this week. But I wasn’t there. Let’s play a game. Can you tell the names of all the animals you saw this week?” Once children responded, the experimenter prompted them with follow-up questions (“Can you tell me more?”; “What are some other animals you saw?”) until the child was done responding. Children’s responses were audio recorded and also written down by the experimenter in the exact order they were stated.

### Scoring

#### Number of animals recalled

The number of animals that children recalled were scored by counting the number of unique animals they recalled in total regardless of the level of detail they provided for each response. For example, whether a child recalled a general animal name (e.g., “zebra”) or a more specific animal name (e.g., “Grevy’s zebra”), either response would count for one point. Five response types were not included in this score: (a) any repeated animals (only the first time that animal was mentioned was counted), (b) any responses that were not informative with regard to specific animal identity (such as “bunch of cute animals”), (c) intrusions (animals that do not exist at the zoo such as “belugas”), (d) animals for which we cannot verify when it may have been seen (i.e., animals that cannot be located in one area, because they roam freely outdoors, like “chipmunks”) and (e) animals found in multiple locations at the zoo for which the child’s response does not allow us to distinguish between the different animals of the same type (e.g., the child only said “hippo” but the zoo has two hippopotamuses, “River Hippopotamus” and “Pygmy Hippopotamus,” which can be found in two different locations at the zoo). The number of cases in which response types (a) to (e) occurred is listed in Table 1. These responses were not included in the primary analysis reported below. In Additional file 1: Result 2, we present analyses with response type (e) included and demonstrate that this does not affect the pattern of results.

**Table 1:** Descriptive statistics and examples of excluded animal responses

Response type	4–5-year-olds		6–7-year-olds		8–10-year-olds		Example responses
	*M*	SD	*M*	SD	*M*	SD	
Repeats or not informative	0.27	0.50	0.43	0.77	0.58	0.89	Repeat: “rhino, rhino” Not informative: “bunch of cute animals”
Intrusions and cannot verify animals	0.98	1.53	0.74	1.28	1.13	1.43	Intrusion: “black and white whale”; animal not found at the zoo Cannot verify: “chipmunks” which cannot be pinpointed to only one area at the zoo
Multiple locations animal (for which child’s response does not allow us to distinguish animals of the same type)	1.87	1.66	2.74	2.15	3.08	2.18	“Tigers”; Two types of tigers can be found in two different locations at the zoo^a^

This table includes the means and standard deviations for the number of responses that were not included in the primary analysis. See Additional file 1: Result 2 for analysis with the “multiple locations animal” responses included

^a^In this example, the response “tiger” does not allow us to differentiate from the two different types of tigers at the zoo, and thus, this type of response was excluded from the primary analyses described in the main text (but see Additional file 1: Results 2). However, if a child’s response *did* allow us to differentiate animals of the same type, then responses were included in the primary analyses. So, if a child said, “Sumatran tiger, …, Amur tiger,” then both responses *would* be included in the primary analyses (each of the two tiger responses receives a free-recall point) since the child’s response allows us to distinguish tigers located in multiple locations at the Zoo. If a child said, “Sumatran tiger, …., tiger,” then, again, both tiger responses would be included in the primary analyses based on the rule described in the main text “Special Cases, Case Type 2”

##### Scoring special cases

Most animals at the zoo were unique, such that we could determine from a “general” animal name where it was located at the zoo. So, if a child recalled only a general animal name (e.g., “fox”), we could determine which animal they were referring to (e.g., “arctic fox”), because only one animal of that kind existed at the zoo. However, we created rules for special cases in which a child stated both the “general” name for an animal and the specific name for the animal during free recall. In Case Type 1, both a general and specific animal name was included in the response, for an animal for which there is only one kind at the zoo. In this case, a point was only given for the mention of the specific animal and the general animal would be considered a repeat (no additional point given). For example, if during recall, the child said, “arctic fox,” and then later during recall said “fox,” the second mention of the fox was considered a repeat. In Case Type 2, both a general and specific animal name was included in the response, but the general animal (e.g., hippo) is located in more than one area of the zoo. In this case, if a child recalled a general animal name that we know is located in multiple locations at the zoo, and also recalled a specific type of that animal, two separate points would be given to that child. This variation to the rule gives children the benefit of the doubt, considering that the child would not be penalized for recalling a general animal that is in multiple locations. For example, the zoo has two types of tigers—the “Amur tiger” and the “Sumatran tiger.” If a child’s recall response included “tiger,” as well as “Amur tiger,” the child would receive a point for each of the two responses on the basis that a general mention of “tiger” could have been a reference to the “Sumatran tiger.” These rules were created a priori and applied to all of the animals the children recalled ensuring consistent scoring among all participants.

#### Temporal clustering

Each location children visited was labeled with a numerical index which represented the order in which they attended that location during the week. For example, if a particular child’s predetermined schedule (confirmed by the counsellor checklist) stated that they visited the Rainforest, Kids Zoo, Americas and then Savanna locations, in that order, then the Rainforest was assigned location number 1, Kids Zoo was assigned location number 2, Americas was assigned location number 3 and Savanna was assigned location number 4. The examples in this paragraph only list a handful of locations to illustrate the procedure. Across the week (Monday through Friday) children visited on average 10.25 locations (SD = 1.50; range: 6 to 13, depending on the week and camp group) that included animals. Daily averages were 2.12, 2.51, 2.14, and 2.38 locations visited on Monday through Thursday, respectively, and an average of 1.10 locations on Friday (prior to the mid-day testing session). Any locations or activities on the schedule that did not include visiting animals were not assigned an index.

In order to examine the temporal organization of each child’s recall responses, location indices were determined for each child, based on their particular schedule over the course of the week. This allowed us to convert their sequence of animal responses during free recall to a sequence of numerical location indices. These served as serial position labels for our temporal clustering analyses. In other words, each animal the child recalled was labeled with the corresponding location index, which reflected the order that they saw the animal during the week. As mentioned above, the serial ordering for different camp groups differed from week to week, as exhibits were not always visited in the same order from group to group.

To give a concrete example of the serial position labeling of response sequences, imagine a child that visited three locations in succession: “Savannah” (location 1, containing a lion), “Americas” (location 2, containing a flamingo), and “Eurasia” (location 3, containing a snow leopard and red panda). For this child, the recall sequence “lion, flamingo, snow leopard” would be coded as “1, 2, 3,” but “snow leopard, lion, flamingo” would be coded “3, 1, 2.” Animals from the same location were given the same location index. Thus, if the child in this example recalled “snow leopard, lion, flamingo, red panda,” this would be coded “3, 1, 2, 3.”

In order to characterize temporal clustering, we calculated a *same-context score* for each child defined as the number of times the child successively recalled animals from the same location. In other words, the same-context score indicated the total number of consecutive values in a recall sequence that have the same location index. The recall sequence “1, 2, 3, 3” would have a same-context score of 1 (due to the “3, 3” in the sequence). The recall sequence “1, 2, 3, 3, 3” has a same-context score of 2 (with “3, 3, 3” representing two consecutive same-context transitions). Finally, the recall sequence “1, 2, 2, 2, 3, 3” has a same-context score of 3 (with 2 points for “2, 2, 2” and another point for “3, 3”).

## Results

We begin by reporting the primary analysis of overall recall performance (in terms of total number of animals recalled) and temporal clustering (in terms of same-context scores). Additional file 1: Results 1 presents secondary analysis with outlier responses removed, and Additional file 1: Results 2 presents secondary analysis including responses that could be associated to more than one location. In both cases, the same pattern of results was obtained as that reported in the primary analysis.

### Number of animals recalled

On average, 4- to 5-year-olds (*M* = 3.27, SD = 3.00) recalled fewer animals than 6- to 7- year-olds (*M* = 7.28, SD = 4.64), and 6- to 7-year-olds recalled fewer animals than 8- to 10- year-olds (*M* = 14.13, SD = 8.21). The assumption of homogeneity of variances was violated, as assessed by Levene’s test for equality of variances (*p* < 0.001), and so we report the Welch analysis of variance (ANOVA); patterns do not change with the standard ANOVA. There were significant age group differences in the number of animals recalled, Welch’s *F*(2,87.19) = 44.32, *p* < 0.001. Post hoc analysis using the Games–Howell test showed that all three age groups were different from each other (all *p*s < 0.001). The mean difference in number of animals recalled between 8–10-year-olds and 4–5-year-olds was 10.87 (95% CI [7.93, 13.80]), between 8–10-year-olds and 6–7-year-olds was 6.86 (95% CI [3.70, 10.02]), and between 6–7-year-olds and 4–5-year-olds was 4.01 (95% CI [2.07, 5.95]). (See Additional file 1: Figure S1-1 for a frequency distribution of total animals recalled for each age group.)

### Temporal clustering: same-context score

All three age groups showed evidence of temporal clustering as measured by the same-context score, and this measure increased in magnitude as age increased (4- to 5-year-olds: *M* = 0.80, SD = 1.27; 6- to 7-year-olds: *M* = 1.34, SD = 1.94; 8- to 10-year-olds: *M* = 4.21, SD = 3.98). These mean values will be called the *observed same-context score* for each age group.

**Fig. 1:**
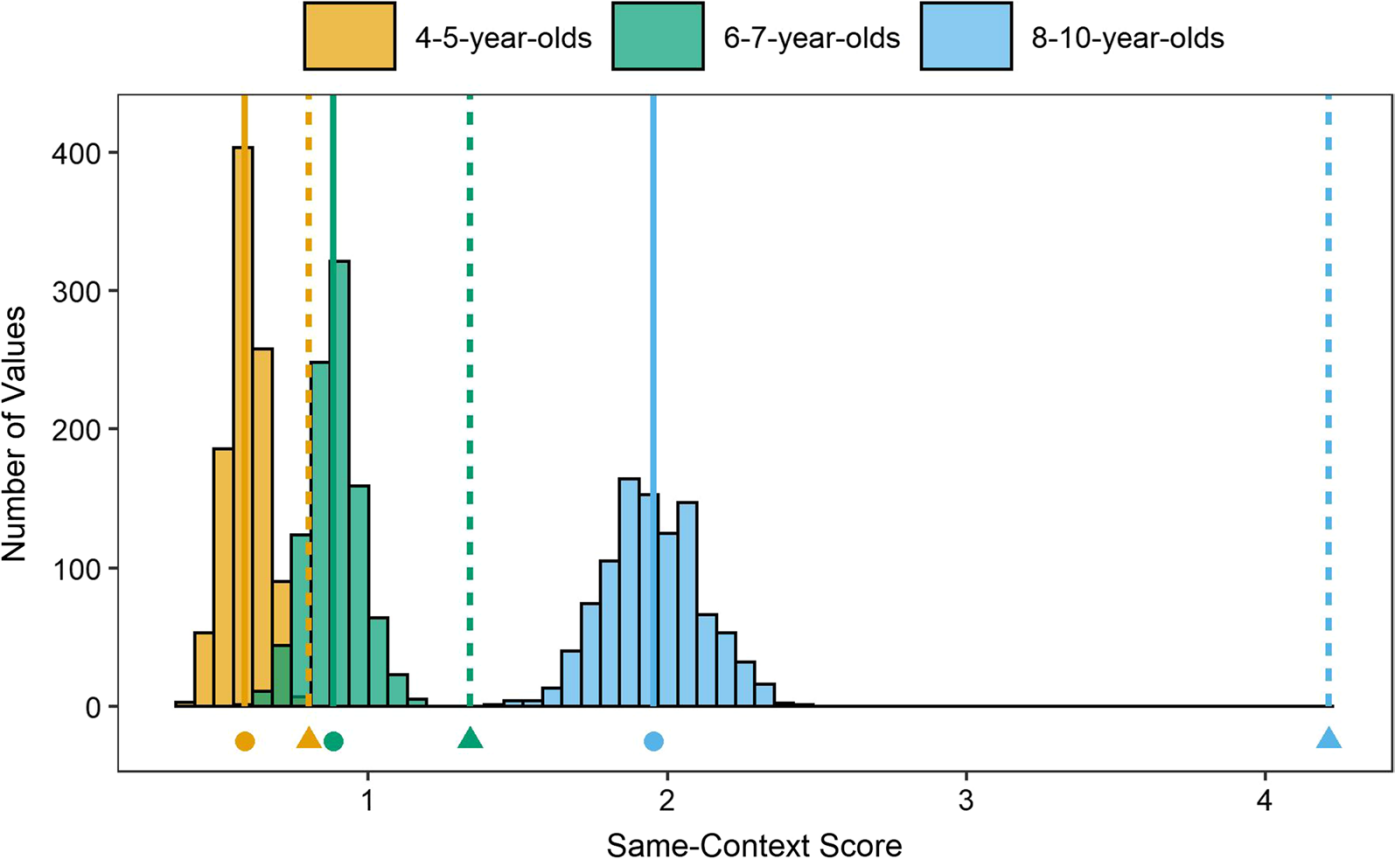
Histogram of permutation distributions and observed same-context scores for each age group. *Note.* Solid vertical lines (and circle symbols) indicate the mean of the permutation distribution. Dashed vertical lines (and triangle symbols) indicate the observed same-context score for each age group

A permutation analysis was conducted to determine the statistical significance of the observed same-context scores. This analysis involved permuting the order of each participant’s responses and recalculating the same-context scores on the permuted response sequences. As such, this analysis naturally accounts for differences in the number of responses across participants and groups. Thus, permutation analysis ensures any observed effects are not confounded with other aspects of behavioral performance. A *permutation distribution* of same-context scores was created for each age group, using a technique similar to past studies (e.g., Miller et al., 2013a, 2013b). To create a permutation distribution, we randomly scrambled the order of recall responses for each participant, calculated the same-context score using this permuted response sequence, and then averaged the scores across participants in a particular age group. This process was repeated 1000 times for each age group, yielding a distribution of 1000 *permuted same-context scores* reflecting chance-level performance. The *observed same-context* score was then compared to the permutation distribution; the proportion of permuted same-context scores exceeding the observed same-context score is interpretable as a *p* value. Figure 1 shows the permutation distribution for each age group as a histogram (1000 samples per age group), with the observed same-context score for that age group indicated with a dashed vertical line. For each age group, the observed same-context score was higher than all 1000 permutation values, indicating reliably above-chance same-context clustering (all *ps* < 0.001).

This analysis revealed substantial temporal clustering in all age groups. But are there age group differences in the amount of clustering? Visually inspecting Fig. 1, we see that the distance between the permutation distribution and the observed same-context score for the youngest age group is smaller than that for the oldest age group. Specifically, the difference between each age groups’ permuted mean (solid vertical line in Fig. 1) and observed mean (dashed vertical line in Fig. 1) increased in magnitude with age: The absolute and relative difference was 0.21 or 36%, 0.46 or 52%, and 2.26 or 115%, for 4–5-, 6–7- and 8–10-year-olds, respectively.

An analysis using the permutation distributions more formally showed that there is a substantial increase in the degree of temporal clustering across age groups. We used each age group’s permutation distribution to calculate a standardized same-context score for each participant (i.e., a z-score). To do this, we first calculated the mean and standard deviation for each age group’s permutation distribution. We then normalized the observed same-context score for each participant in each age group by subtracting the mean value of the permutation distribution, and dividing by the standard deviation. An ANOVA revealed significant age group differences in these standardized same-context scores (a main effect of age group, *F*(2,141) = 3.69, *p* = 0.027, *η*_*p*_^2^ = 0.05). The standardized scores for 8–10-year-olds was greater than both younger age groups (*p*s < 0.05; *p* = 0.014 for 4–5-year-old comparison; *p* = 0.034 for 6–7-year-old comparison); the two younger groups did not differ from each other (*p* = 0.73). With stringent Bonferroni correction, the difference between the 4–5-year-old and 8–10-year-old groups remained significant (*p* = 0.04); but the difference between 6–7-year-olds and 8–10-year-olds was no longer significant (*p* = 0.10).

Overall, all age groups showed evidence of same-context clustering, with this effect exhibiting itself more robustly for the oldest age group relative to the youngest age group. This effect persists after normalization with an age-specific permutation distribution, suggesting that it is not an artifact of differences in recall performance between the groups.

#### Post hoc analyses: relation between recall performance and temporal clustering

Partial correlation analysis, controlling for age in months, showed that individual differences in temporal clustering (measured with same-context scores) was positively correlated with number of animals recalled, *r*_partial_(141) = 0.759, *p* < 0.001. This significant positive correlation was also apparent for each age group considered on its own (4–5-year-olds: *r*(43) = 0.843, *p* < 0.001; 6–7-year-olds: *r*(45) = 0.674, *p* < 0.001; 8–10-year-olds: *r*(50) = 0.790, *p* < 0.001).

## Exploratory analyses

### Adjacent context scores

A hallmark of temporal organization in list-based free-recall tasks is that the effect weakens gradually as the temporal spacing of two items (their “lag”) increases, reaching a baseline asymptote by about lag ± 5. A set of exploratory analyses examined whether the same was true for temporally adjacent locations on children’s schedules. These included a custom analysis examining the likelihood of recall transitions to adjacent contexts in the forward and reverse direction, as well as two traditional measures of temporal organization: a percentile-rank temporal organization score and a lag-based conditional response probability (lag-CRP) analysis. None of these exploratory analyses revealed evidence of temporal organization, as described in Additional file 1: Results 3. In other words, the temporal organization we observed was limited to clustering of animals seen within a particular location, and did not spread to neighboring locations. This may reflect the fact that each location encompasses the equivalent of several serial positions in a standard list-based task. In standard tasks, recall transitions of this distance do not reliably show temporal organization.

### Primacy and recency effects

**Fig. 2:**
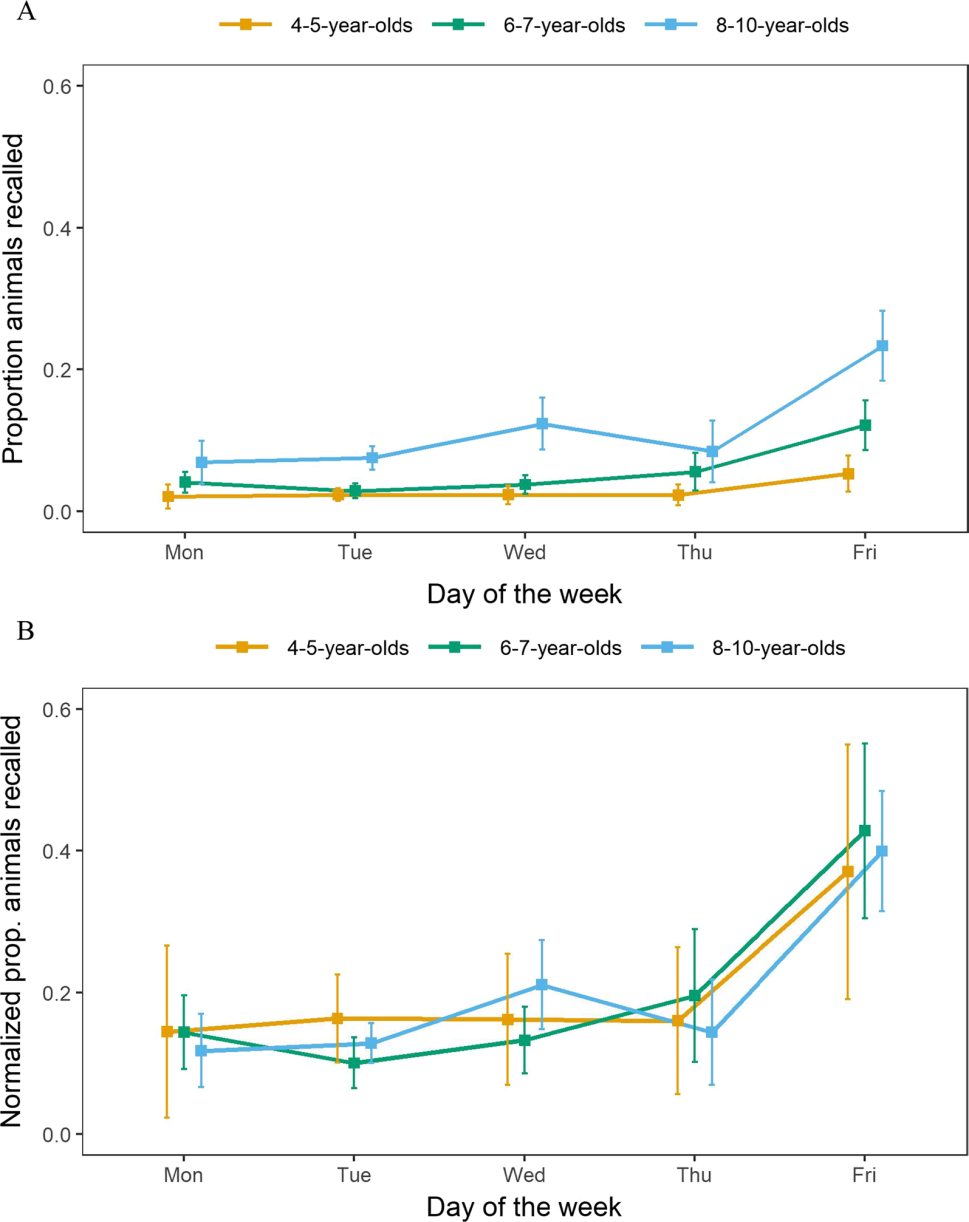
Serial position analyses. *Note.* Panel **A**: Serial position analysis of proportion of animals recalled from each day of the week. Panel **B**: When recall proportions are normalized relative to total number of animals recalled, all three groups show similar proportional recency effects. Each age group was horizontally offset from the other groups to prevent overplotting; error bars represent standard errors

Another form of temporal structure observed in free recall is the memory advantage seen for items from the beginning or end of a study list. We explored whether the children showed memory advantages for animals encountered early in the week (i.e., a primacy effect) or late in the week (i.e., a recency effect). In a typical laboratory-based free-recall task there is only 1 possible item that could be correctly recalled for each serial position. For our naturalistic study, we treated the day of the week (i.e., Monday, Tuesday, etc.) as analogous to serial position, except that multiple items (i.e., animals) were encountered each day. Thus, each serial position value was calculated as a proportion; the number of animals from that day that were recalled, divided by the total number of animals seen that day (based on child’s schedule). This analysis, depicted in Fig. 2A, shows that while all groups showed recency effects, none showed a clear primacy effect.

The recency effect depicted in Fig. 2A is weakest for the youngest age group and becomes progressively larger for the older groups. However, given the substantial differences in overall recall performance (i.e., overall number of animals recalled) between the age groups, we conducted a follow-up analysis (Fig. 2B) that normalized each serial position curve relative to overall recall performance. Specifically, we divided each proportion value from Fig. 2A by the sum of the recall proportions across the 5 days, thus ensuring that the set of 5 normalized recall scores summed to 1. This normalization allows us to examine the magnitude of the recency advantage relative to recall performance for the other 4 days. With this normalization, it becomes apparent that the three age groups show recency effects of equivalent magnitude.

We quantified the primacy and recency effects using difference scores (primacy: difference of Monday proportion recalled and Tuesday proportion recalled; recency: difference of Friday proportion recalled and Thursday proportion recalled). We carried out a set of one-way ANOVAs on the unnormalized and normalized difference scores. For the primacy effect, neither the unnormalized or normalized difference scores showed a main effect of age group (both *p*s > 0.5). A set of follow-up t tests showed that none of the primacy difference scores for any of the age groups were reliably different from zero (age 4–5, *p* = 0.79; age 6–7, *p* = 0.14; age 8–10, *p* = 0.71). For the recency effect, a set of one-way ANOVAs revealed a main effect of age group on difference score for the unnormalized difference scores (*F*(2,141) = 5.88; *p* < 0.005) but not for the normalized difference scores (*F*(2,141) = 0.08; *p* = 0.93). A set of follow-up t tests revealed that the recency difference scores were reliably different from zero for the two older groups (4–5-year-olds, *p* = 0.06; 6–7-year-olds, *p* = 0.002; 8–10-year-olds, *p* < 0.001).

#### Influence of previous interview question

The findings from this exploratory question are presented in Additional file 1: Results 4.

## Discussion

In the present study, events occurred over an extended period, there were multiple types of naturally occurring delays and distractors, both between events that would later be recalled, and between study (experiencing) and test (recalling). Further, children experienced these events unaware of what would be later tested. Even under these challenging conditions, temporal clustering was above that expected by chance for children as young as 4–5 years old. Our finding in children parallels previous studies reporting temporal contiguity in adults over longer timescales than typically examined in the laboratory (Mack et al., 2017; Uitvlugt & Healey, 2019). Further, our approach was sensitive enough to capture developmental change: Older children showed more temporal clustering than younger children. Thus, this fundamental memory principle is functional in early childhood, but shows continued change early in the lifespan, and aligns with previous work showing weakened temporal clustering late in the lifespan (e.g., Golomb, et al., 2008; Kahana et al., 2002). Given that individual differences in degree of temporal clustering predicts individual differences in the number of items recalled in adults (Sederberg et al., 2010), it is possible that older children recall more events than younger children because the mechanisms underlying temporal clustering are more mature and contribute to better recall. Post hoc analyses showed that individual differences in same-context scores was positively correlated with individual differences in children’s recall success, even when controlling for age. Although we should be mindful that correlation does not equate to causation, our results are consistent with previous studies showing positive correlations between temporal contiguity and recall performance (see Healey et al., 2019), and suggest that even in children, temporal clustering effects are meaningful and an important part of the search process.

Current theories of memory search propose that one constructs a retrieval cue to probe stored memories in a task-dependent manner (Becker & Lim, 2003; Hintzman, 1984; Polyn et al., 2009; Raaijmakers & Shiffrin, 1981). Organizational effects arise in these models when retrieved information related to a remembered item is incorporated into the retrieval cue, influencing the next recall response (Healey et al., 2019). If that retrieved information specifies the temporal context of the remembered item, this will support the remembrance of temporally proximal neighbors, and temporal organization will likely be observed. Our findings suggest that children as young as 4 and 5 years old, like adults, encode information about temporal context automatically (see Healey et al., 2019). However, the memory traces stored by 4–5-year-olds may have weaker temporal structure than those of 8–10-year-olds, causing remembered events to cluster less reliably with regards to their temporal proximity. At the same time, our youngest children may have been less efficient at constructing and iteratively adapting retrieval cues to guide search, which may also be the case in older adults (Diamond & Levine, 2020). Future work to disentangle both possibilities would extend the small literature that have reported on temporal clustering in children’s memory search (e.g., Kelly & Beran, 2021).

In terms of our exploratory serial position analysis, we found a recency effect, but no primacy effect. Primacy can be due to selective processing of early items in a sequence (Atkinson & Shiffrin, 1968; Tan & Ward, 2000) aided by additional rehearsal of early items. Here, children likely reminisced about camp events at home throughout the week. There is no reason to believe that reminiscing would have focused on early elements the way a more intentional rehearsal strategy might. Thus, a lack of primacy effect is not surprising. It is also consistent with studies showing more robust recency than primacy effects in the laboratory in children (Glidden, 1977; Jarrold et al., 2015; Kelly & Beran, 2021), and recency but no primacy effects in real-life events for adults (Uitvlugt & Healey, 2019; but see Diamond & Levine, 2020, for different patterns). Further, our approach has features in common with continual-distractor free-recall tasks for which there are robust long-term recency effects (Bjork & Whitten, 1974) and our findings are consistent with models which posit that a recency effect will be observed when events are spaced throughout a temporal interval targeted for recall (Brown et al., 2007; Sederberg et al., 2008).

Two caveats are noted, both consequences of uncontrolled real-world research. First, events that were proximal in time were also proximal in space. This applies to other naturalistic studies (see discussions by Diamond & Levine, 2020; Healey et al., 2019) and is important because, in addition to temporal clustering, there is evidence of spatial clustering (adults tend to recall in succession landmarks that are geographically proximal; Miller et al., 2013a, 2013b) and semantic clustering (items that are semantically related tend to be recalled consecutively; Manning & Kahana, 2012). In the laboratory, individual items within the list can be designed to measure one type of clustering, but in the real world, events naturally co-occur and two events experienced close in time are likely to be experienced close in space, and often are, or become, semantically related. It is out of the scope of the present research to disentangle the multiple forms of clustering potentially influencing our same-context scores.

A second caveat regards our exploratory examination of recall transitions between temporally adjacent zoo contexts. Typical lag conditional response probability functions show a gradual drop in transition likelihood as temporal distance increases, with a forward asymmetry (transition to an adjacent item in the forward direction). While our same-context analysis showed temporal organization, our adjacency analysis (and lag-CRP analysis; Additional file 1: Results 3) did not show temporal organization between one location and its temporal neighbor (location visited previously or next on child’s schedule). Other naturalistic studies also show temporal organization limited to events occurring within the same time bin (Moreton & Ward, 2010), or limited to immediately adjacent time bins (Uitvlugt & Healey, 2019). However, these findings contrast with Diamond and Levine (2020) who showed temporally graded and forward asymmetric effects with younger and older adults for naturalistic events, and with studies showing forward asymmetry with children in the laboratory (Jarrold et al., 2015; Lehmann & Hasselhorn, 2012). The discrepancy likely involves the granularity with which temporal structure was coded. Children were not tracked within zoo locations, which limited our temporal precision (any possible adjacency effects within a location were counted as same-context transitions). In future work, children could wear cameras to track the exact order animals were encountered within exhibits.

In conclusion, in our sample of participants, children’s memory search showed universal memory principles that parallel those seen in the adult memory system, while also showing continued change between early and late childhood. Further, we found such effects with real-world events.  Thus, this work demonstrates the robustness and ecological validity of temporal structure as a fundamental organizing principle of memory (Healey & Kahana, 2014). It also provides empirical evidence to help bridge the adult and developmental literatures, and the laboratory-based and naturalistic memory literatures.

## Supplementary Information

**Additional file 1. Supplemental Analyses:** Results 1 shows analyses with outliers removed; Results 2 shows analyses including additional animal responses; Results 3 shows analyses of adjacent context scores; Results 4 shows results about the previous interview question.

## Data Availability

Analyses, permutation scripts, figure scripts and anonymized data are openly available on the Open Science Framework at osf.io/fskxh.
