# Supplementary material for: Children’s memory “in the wild”: examining the temporal organization of free recall from a week-long camp at a local zoo
Source: Cogn Res Princ Implic. 2023 Jan 25;8:6. doi: 10.1186/s41235-022-00452-z (PMC9873889; doi:10.1186/s41235-022-00452-z)
Supplement: Supplementary file 1 — Additional file 1. Supplemental Analyses: Results 1 shows analyses with outliers removed; Results 2 shows analyses including additional animal responses; Results 3 shows analyses of adjacent context scores; Results 4 shows results about the previous interview question. [file 41235_2022_452_MOESM1_ESM.docx]

**Supplemental Results 1**

It is debated whether, and how, outliers should be removed in analyses (see Osborne & Overbay, 2004). For transparency, we include here analyses with outliers removed. In Figure S1-1, the two participants that recalled approximately 40 animals during recall can be considered outliers. Their scores were not due to reporting error; they are simply cases in which children recalled numerous animals. Below is the parallel analysis reported in the main manuscript, with these two participants removed.

**Figure S1-1**

*Frequency Distribution of Total Animals Recalled for All Participants*

**
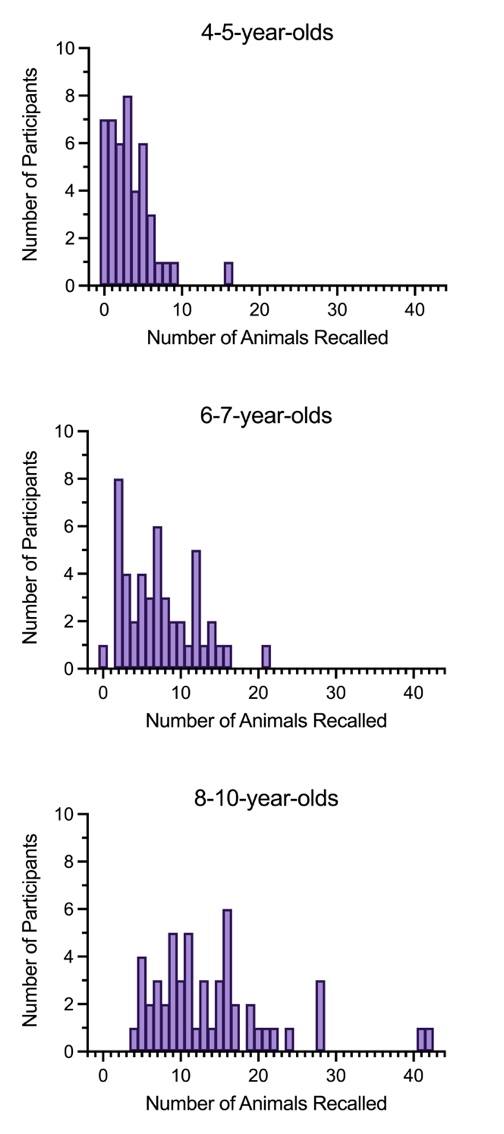
**

**Number of Animals Recalled**

After removing outliers, on average, 4-5-year-olds (*M =* 3.27, *SD =* 2.995) recalled fewer animals than the 6-7-year-olds (*M =* 7.28, *SD =* 4.642), and 6-7-year-olds recalled fewer animals than 8-10-year-olds (*M =* 13.04, *SD =* 6.197). An ANOVA revealed age-related improvements in the number of animals recalled, Welch’s *F*(2, 87.53)= 52.19, *p*<0.001. Follow-up with the Games-Howell test showed all three age groups performed differently from each other (*p*s < .001).

**Temporal Clustering: Same-Context Scores**

Figure S1-2 shows the permutation distribution for each age group as a histogram, with the observed same-context score for that age group indicated with a dashed vertical line. For each age group the observed same context score was higher than all 1000 permutation values, indicating reliably above-chance same-context clustering (all *ps*<0.001).

**Figure S1-2**

*Permutation Distribution and Observed Context Score for Each Age Group, Excluding Outlier Responses*


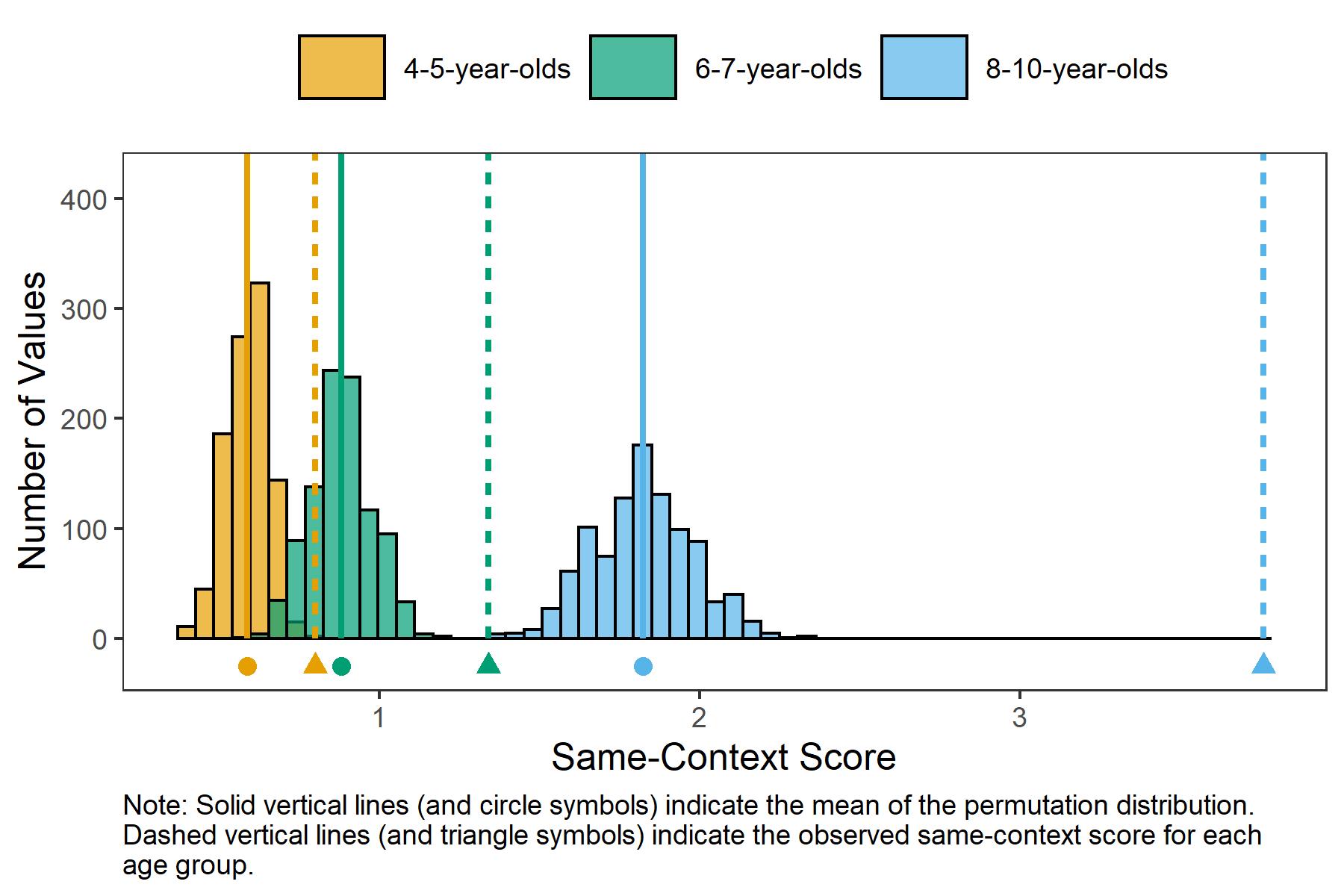


**Additional Note**

To further assess the robustness of reported effects we completed parallel analysis focusing on children who recalled at least 4 animals. This reduced the sample size substantially (*n*=17, 34, and 52, for 4-5, 6-7 and 8-10-year-olds, respectively). Still, the same pattern of results for ‘Number of Animals Recalled’ and ‘Temporal Clustering’ remained.

**Supplemental Results 2**

As described in the main text, the primary analysis was conducted excluding responses in which the recalled animal could have been encountered in more than one location at the zoo (listed in manuscript as response type ‘e’; see Table 1). Here we report analyses with these responses included.

**Number of Animals Recalled**

On average, 4-5-year-olds (*M =* 5.13, *SD =* 4.07) recalled fewer animals than the 6-7-year-olds (*M =* 10.04, *SD =* 5.69), and 6-7-year-olds recalled fewer animals than 8-10-year-olds (*M =* 17.21, *SD =* 8.86). An ANOVA revealed age-related improvements in the number of animals recalled, Welch’s *F*(2, 90.12)= 41.81, *p*<0.001. Follow-up with the Games-Howell test showed all three age groups performed differently from each other (*p*s < .001).

**Temporal Clustering: Same-Context Scores**

For every recalled animal that could have been encountered in more than one location (for which the child’s response does not allow us to distinguish which location the animal was located; e.g., child only said ‘hippo’ and there are two types of hippos at the zoo located in different locations), we randomly selected one of the multiple locations using an online random number selector (https://andrew.hedges.name/experiments/random/). This randomly selected number served as the location index for that child’s recall of that animal. For example, if the recall sequence was “1, 4, 7, 8/6/4, 6, 10” with “8/6/4” representing the possible location indices for that particular animal, we entered the location numbers 8, 6 and 4 into the random number selector and used the randomly selected number to create a new recall sequence for that child. Suppose the randomly selected number was “6” (from the three options “8/6/4”), then the recall sequence would now be “1,4,7,6,6,10”.

This was done for all participants that mentioned animals from multiple locations (for which the child’s response does not allow us to distinguish which location the animals were located) and the newly generated recall sequences were used to calculate the observed same-context scores and conduct the permutation distribution analysis. We found that all age groups showed evidence of clustering; observed same-context scores were higher than permutation distributions, *ps*<0.001 (see Figure S2-1). For the 4-5-year-old group the observed same context score was higher than 999 of the 1000 permutation values, and for the two older groups this was the case for all 1000 permutation values, indicating reliably above-chance same-context clustering (all *ps*<0.001).

**Figure S2-1**

*Permutation Distribution and Observed Context Score for Each Age Group, Including Multiple Location Animals*


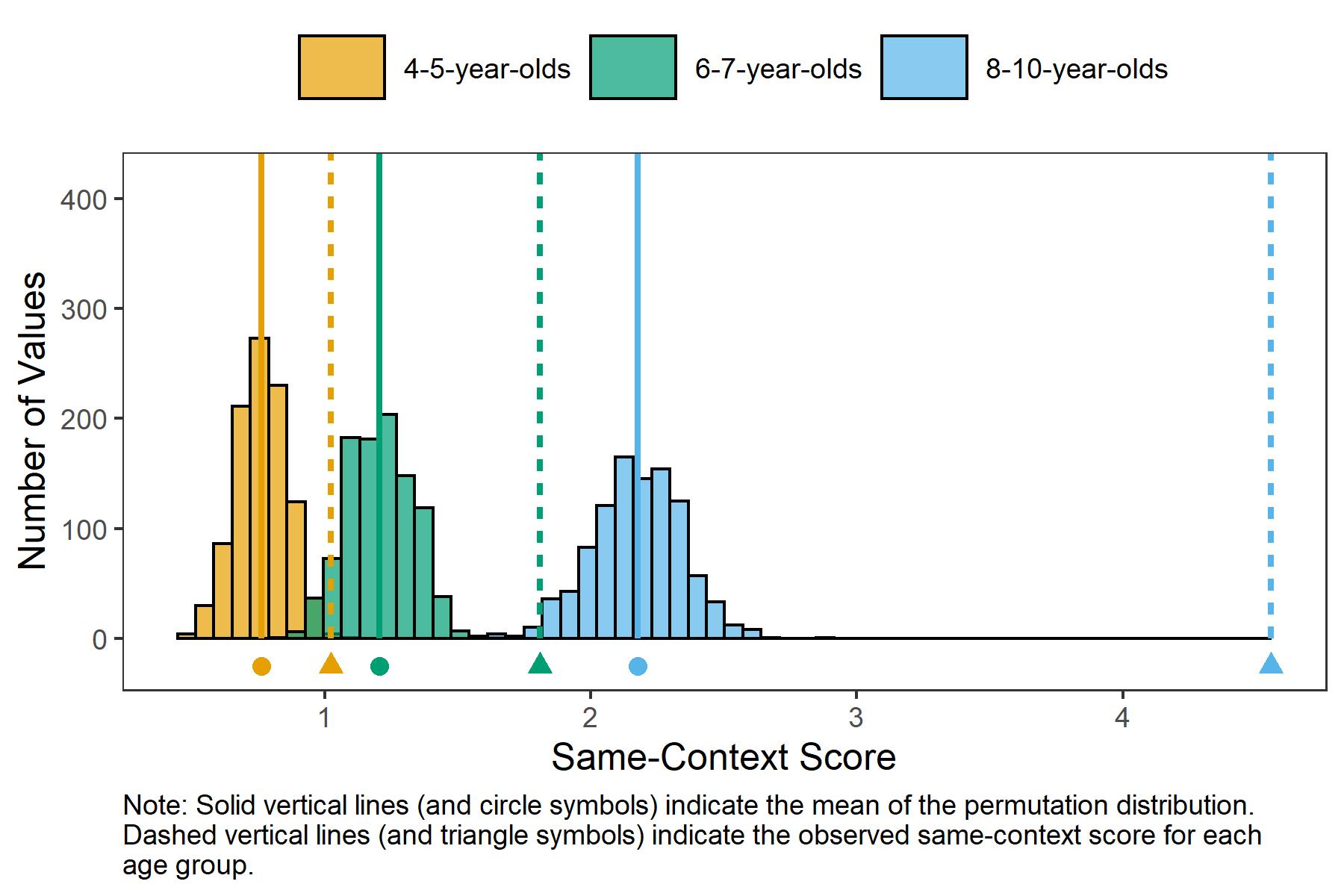


**Supplemental Results 3**

We explored whether temporal clustering effects would spread to adjacent locations in the child’s schedule. Adjacent context scores were computed as the number of times the child successively recalled animals from one location to a neighboring location on their schedule, in the forward and reverse direction. Note that although adjacent locations were temporally proximal (based on the child’s schedule) they may not have been spatially proximal. The forward-adjacent score indicated the total number of consecutive values in a recall sequence that have +1 location index, and the reverse-adjacent score indicated the number of consecutive values in a recall sequence that have -1 location index. For example, the recall sequence “1, 2, 3, 6, 5” would have a forward adjacent context score of 2 (due to the “1, 2” and “2, 3” in the sequence) and would have a reverse adjacent context score of 1 (due to the “6, 5”). As a reminder, a location index here is not equivalent to serial positions in lab-based studies since in our study there were numerous animals in any one location whereas in typical studies of lag-CRP (conditional-response probability; Kahana, 1996) there is a one-to-one correspondence between item and serial position. Further, this score did not consider whether consecutive location indices occurred in the same day or from one day to another (i.e., last location visited on Tuesday and first location visited on Wednesday morning would be given the same score as a transition occurring within the same day).

We conducted the same permutation distribution analyses as reported in the main text except using the forward adjacent context and reverse adjacent context scores, instead of the same context scores. Again each permutation distribution consisted of 1000 samples. For each sample, we randomly scrambled the order of recall responses for each participant, calculated adjacent/reverse context scores using this permuted response sequence, and then averaged the scores across participants in a particular age group. This process was repeated 1000 times for each age group, yielding a distribution of 1000 *permuted forward/reverse adjacent context* *scores* reflecting chance-level performance. The *observed forward/reverse adjacent context* scores were then compared to the corresponding permutation distribution; the proportion of permuted forward/reverse adjacent context scores exceeding the observed forward/reverse adjacent context score is interpretable as a *p*-value. Figure S3-1 shows the permutation distribution for each age group as a histogram, with the observed forward adjacent context score for that age group indicated with a dashed vertical line. The observed forward adjacent context score was only higher than 731, 194, and 190 permutation values (out of 1000 permutation values), for the 4-5, 6-7, and 8-10-year-olds age groups respectively. Figure S3-2 shows the permutation analysis for the observed reverse adjacent context score. The observed reverse adjacent context score was only higher than 886, 831, and 36 permutation values (out of 1000 permutation values), for the 4-5, 6-7, and 8-10-year-olds age groups respectively. Thus, there was no above-chance adjacent context clustering in either the forward or reverse directions (*p*s>0.05).

**Figure S3-1**

*Permutation Distribution and Observed Forward Adjacent Context Score for Each Age Group*


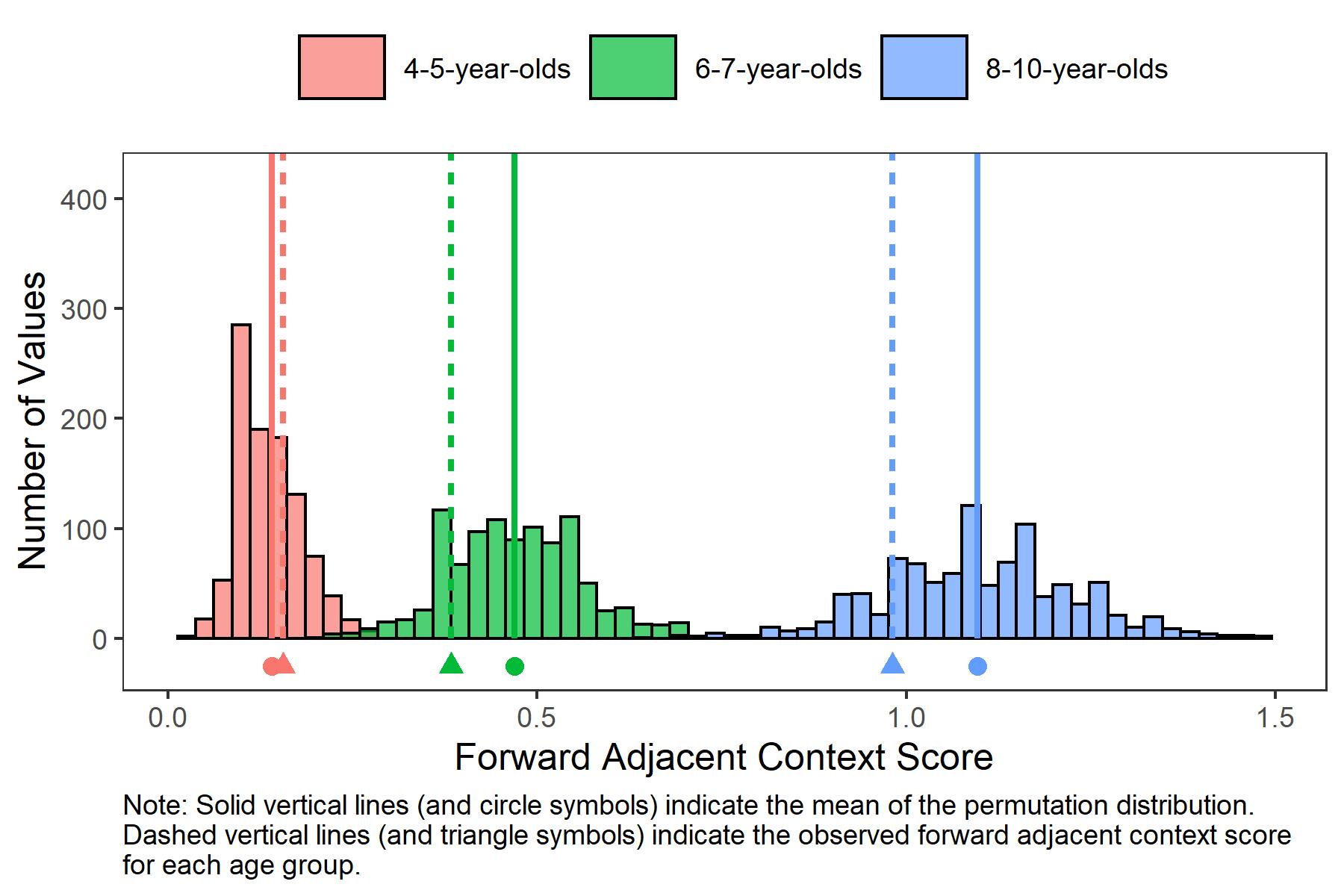


**Figure S3-2**

*Permutation Distribution and Observed Reverse Adjacent Context Score for Each Age Group*


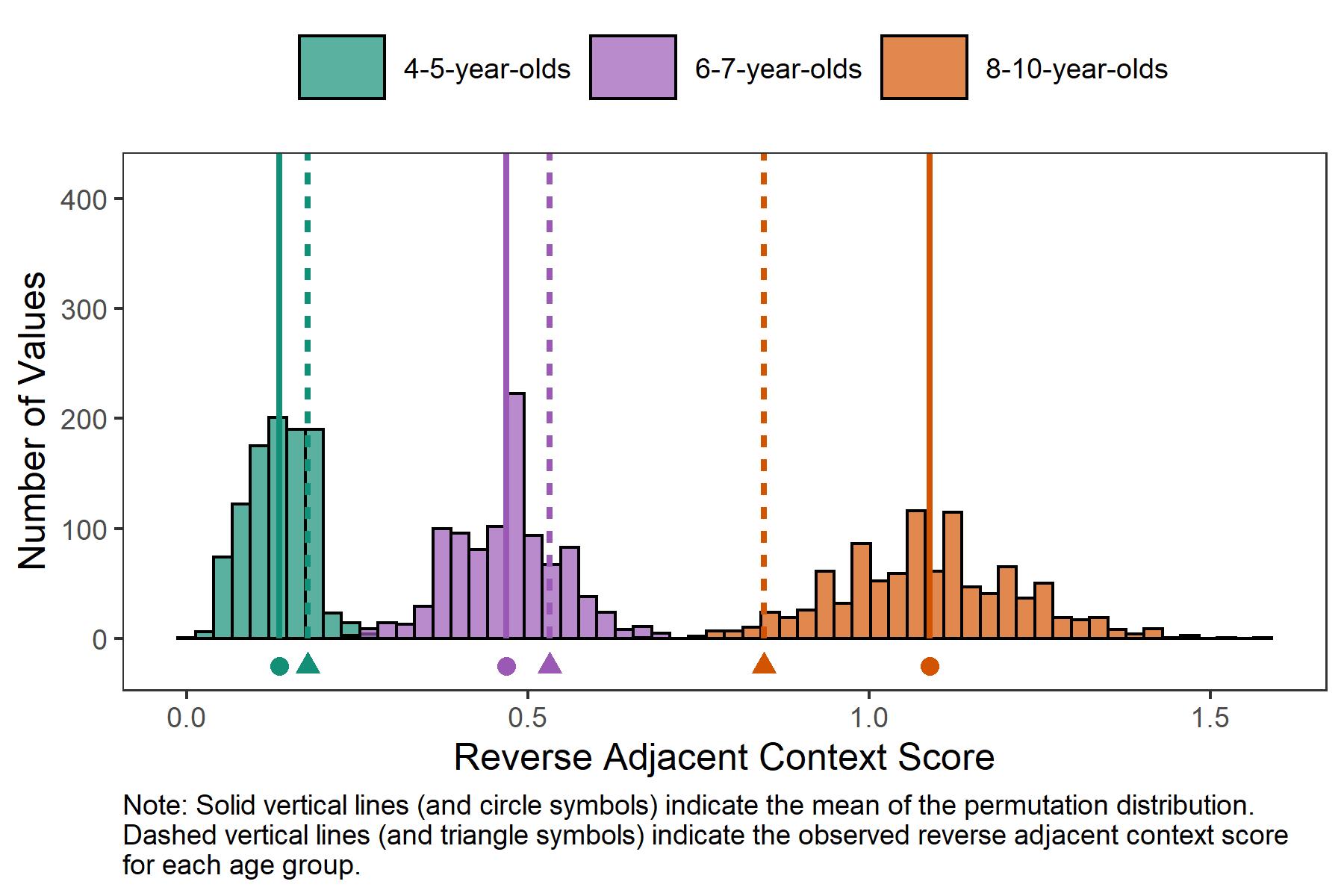


**Lag-CRP and Percentile-Rank Temporal Organization Score**

We also conducted two traditional analyses of temporal organization, a percentile-rank temporal organization score analysis, and a lag-CRP analysis (Polyn et al., 2009; Healey et al., 2018). The results were consistent with the above results for adjacent context scores. These analyses treat the recall sequence as a series of recall transitions *from* one location *to* another location. The locations are labeled by their ordinal position in the overall sequence of locations visited by a given participant over the course of the week, and each recall transition is assigned a lag distance separating the *from* and *to* locations. By design, these two analyses exclude same-context transitions, because in standard list-learning tasks only one item is associated with each position, making a recall transition of lag 0 impossible. The percentile-rank temporal organization score assigns each recall transition a percentile ranking ranging from 0 to 1. This rank indicates the temporal proximity of the two recalled items compared to the set of possible transitions *from* that particular item at that point in the recall sequence. The overall score for a given data set is the average of the percentile ranks assigned to all of the observed recall transitions, with 1.0 indicating perfect temporal organization, and 0.5 indicating chance-level temporal organization. The group means for the percentile-rank temporal organization score were 0.4930, 0.5046 and 0.5045, for 4-5-, 6-7, and 8-10-year-old groups, respectively; none of these scores were reliably different from the chance value of 0.50.

The lag-CRP analyses depicted in Figure S3-3 allow us to visualize temporal organization by calculating the conditional probability of recall transitions of different lag distances. These probabilities reflect the likelihood of observing a transition of a particular lag, conditional on whether that lag was possible (e.g., if the *from* location was the first location visited, transitions with negative lags are not possible for that transition). Reliable temporal organization in a lag-CRP analysis appears as larger probabilities for shorter lag transitions relative to longer lag transitions. This pattern was not apparent for any of the three age groups.

**Figure S3-3**


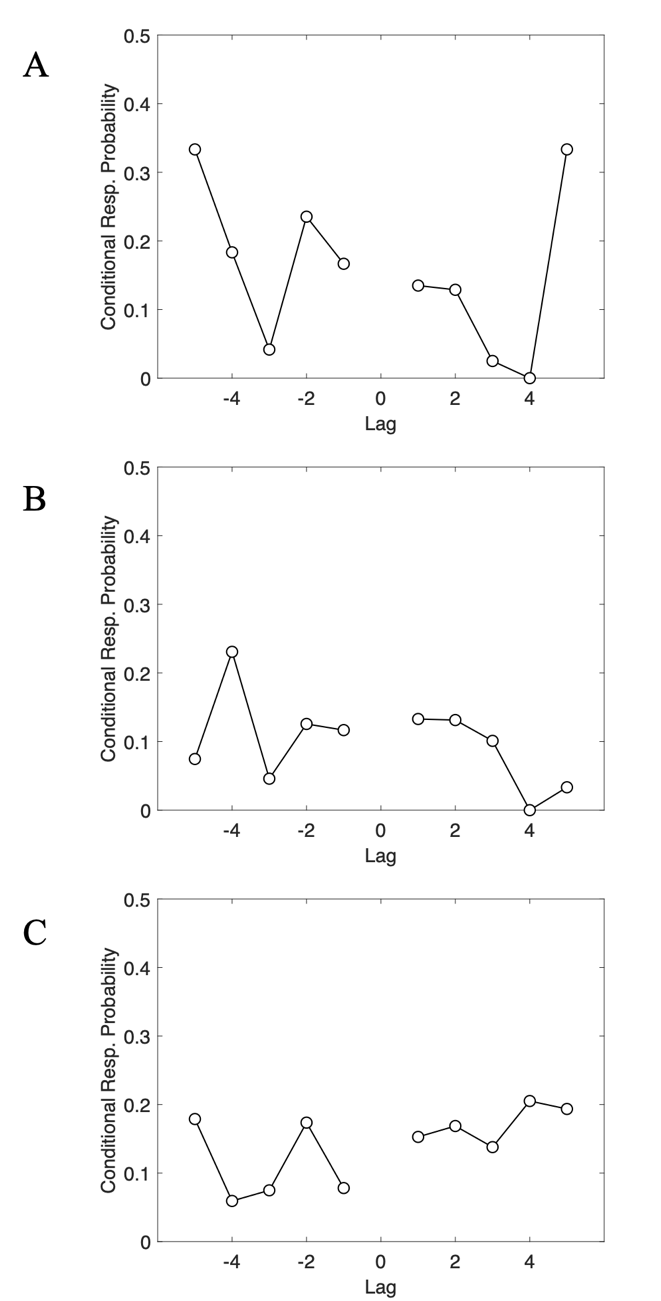
*Lag-CRP for 4-5-year-olds (A), 6-7-year-olds (B) and 8-10-year-olds (C)*

**Supplemental 4**

**Influence of Previous Interview Question*.*** During the test session, prior to beginning the free recall task, participants were asked to tell the experimenter about their visit to their *favourite* animal at the zoo. Thus, the particular animal discussed for this interview question varied for each child (each child selected the animal). To explore whether this may have influenced how children initiated their free recall, we calculated the percentage of children that initiated recall with their favourite animal. We also calculated the percentage of children that either initiated recall with their favourite animal or initiated recall with an animal that was from the same location as their favourite animal. Participants were not included in this analysis if they did not recall any animals (*n*=4), were not asked the favourite animal question (*n*=2), did not specify one favourite animal (*n*=1), or stated a category of favourite animals (*n*=1; favourite animal was ‘the birds’).

The percentage of children for which their favourite animal from the prior interview question was the first animal response in their free recall were 18.42%, 8.70%, and 11.54%, for 4-5-, 6-7-, and 8-10-year-olds, respectively. When we included in addition the children who initiated recall with an animal from the same location as their favourite animal the percentage of children were 52.63%, 32.61%, and 25.00%, for 4-5-, 6-7-, and 8-10-year-olds, respectively. Note for these analyses we used the first animal in their recall, whether or not it was an animal that could have been found in more than one location at the zoo. This did not affect the favourite animal percentages, since location was not involved (we simply counted the number of children for which there was a match between the animal the child stated as their favourite animal in the prior question and the first recalled animal in the free recall). This would impact the percentages for the second set of percentages reported above. When scoring this we decided to give the child the “benefit of the doubt”; Thus, if the first recalled animal was one that could be from multiple locations, we gave the child a point for a match if the favourite animal location from the prior question matched any of the possible locations for the first animal recalled. In other words, we purposefully overestimated the impact of the prior question. In summary, less than 20% of children initiated recall with the animal discussed in the prior interview question. This relatively low percentage may not be surprising- if children already told the experimenter about this animal they may have thought it was not necessary to include it in their first recall. When we also include cases where the child initiated recall with an animal from the same location as their favourite animal, we found that approximately half of the youngest group and approximately a quarter of the oldest group could have been influenced by the prior interview question for the start of their memory search. Although findings should be interpreted cautiously, it seems that older children were less likely to initiate recall with an animal from the same location as their favorite animal, compared to younger children. In other words, it is possible that older children were more independent in their formation of cues to initiate memory search. Still, even with overestimation, only half the youngest group showed evidence of being influenced by the prior test question, and so reliance on external sources to initiate recall did not dominate young children’s recall initiation.
